# The role of the natural and built environment in cycling duration in the Netherlands

**DOI:** 10.1186/s12966-018-0715-z

**Published:** 2018-08-29

**Authors:** Jie Gao, Carlijn B. M. Kamphuis, Martin Dijst, Marco Helbich

**Affiliations:** 1 Department of Human Geography and Spatial Planning, Faculty of Geosciences, Utrecht University, Princetonlaan 8a, 3584 CB Utrecht, The Netherlands; 2 Department of Interdisciplinary Social Science, Faculty of Social and Behavioral Sciences, Utrecht University, Heidelberglaan 1, 3584 CS Utrecht, The Netherlands

**Keywords:** Cycling, Natural and built environment, Municipality, Multilevel regression model, The Netherlands

## Abstract

**Background:**

Cycling for transportation has the potential to contribute to an increase in people’s physical activity levels. A growing body of evidence links the natural and the built environment to cycling. Whereas previous studies were mostly done within one city or one region, the present study covers the whole of the Netherlands, allowing an investigation of whether associations between environmental characteristics and cycling are context-specific. The study examines the extent to which objectively measured natural and built environment characteristics contribute to cycling duration in the Netherlands, as well as the differential effect of environmental characteristics on cycling duration by municipality size.

**Methods:**

Our sample from the Dutch National Travel Survey 2010–2014 comprised 110,027 people aged 20–89 years, residing in 3163 four-digit postal code areas, nested within 387 municipalities across the whole of the Netherlands. Multilevel Tobit regression models were fitted to assess the associations between the natural and the built environment with average daily cycling duration (in minutes), while adjusting for individual and household characteristics. Interaction effects of natural and built environment characteristics and municipality size on cycling duration were also investigated.

**Results:**

Higher address density, more bus stops, and shorter distance from home to the nearest train station were positively related to cycling duration. Respondents were more likely to cycle on days with higher temperatures, less wind, and less precipitation. Interaction tests showed that increased street density and address density were less cycling-promotive in small urban areas compared to medium or large cities. On the other hand, the positive association between number of bus stops and cycling duration was weaker in the largest and medium-sized cities compared to small urban and rural areas.

**Conclusions:**

Interactions suggest that relations between environmental characteristics and cycling duration are context-specific (i.e., dependent on circumstances that differ between highly urbanized and less urbanized areas). Our findings need to be replicated in other countries to gain more insight into the interplay between environmental factors and municipality size.

## Background

Physical activity provides a range of health benefits and reduces the risks of chronic diseases, such as obesity, diabetes, and high blood pressure [1, 2]. Cycling for transportation has the potential to contribute to an increase in people’s physical activity levels [3], and is an environmentally sustainable mode of transportation [4]. As a consequence, national and local governments are eager to promote cycling in order to obtain the associated health benefits [5–7]. Large variations exist in bicycle use between countries. It was estimated that bicycling accounts for about 1–2% of all trips in North America and Australia [8], which is a much lower percentage than in northern Europe: Figures range from a high of 27% in the Netherlands (all ages) to 18% in Denmark (10–84 years) and around 10% in Finland (6+ years), Germany (all ages), and Belgium (6+ years) [9, 10]. However, bicycle use varies not only between countries, but also between areas and municipalities within a country [11]. Although 27% of all trips are made by bicycle in the Netherlands [12, 13], there are substantial variations in the share of short-distance (i.e., up to 7.5 km) bicycle trips between Dutch municipalities; for example, the share is 17% in Heerlen^1^ and nearly 50% in Groningen^2^ [14]. These large variations between municipalities possibly correspond to variations in environmental characteristics, but few studies have examined this [6, 15].

Studies have shown that cycling duration is related not only to individual attributes (e.g., sociodemographic characteristics), but also to the environment in which people live and move around [16–18]. There is empirical evidence that population and address density, land use, building diversity, and urban design (e.g., street network configurations) affect cycling levels [19–23]. The effects of population density on cycling behavior might often be indirect. A higher population density is often required to support a greater diversity in local destinations and to reduce distances between places. Higher population density is found to relate to a higher likelihood of bicycle use [7, 24]. Similarly, land use diversity, characterized by a mixed land-use, brings origins and destinations closer together and shortens trip distances [24–26]. Mixed-use neighborhoods make trips by bike more convenient [27]. Regarding urban design, a cycling friendly street network and infrastructure characteristics may increase the accessibility of different destinations by bicycle. Studies suggest that people who live in neighborhoods that have been designed to be cycling friendly, which are characterized by higher levels of street connectivity, may increase the likelihood of bicycle use [19, 27–30].

In addition to the built environment factors, natural environment characteristics are also thought to be important [4, 29]. Various studies have investigated the effects of weather on daily bicycle use [31–33], and found that sunshine and warm weather increased the probability of commute cycling, and that cold weather and windy weather were inversely associated with cycling [34]. Weather may influence the relation between environmental determinants and bicycle use [35]. Since cyclists are directly exposed to the elements, high or low temperatures and heavy precipitation may make cyclists hesitant to expose themselves for too long. Therefore, the effects of weather may be inherently related to cycling duration.

Even though these studies contributed significantly to our understanding of how natural and built environments are related to cycling behavior, findings appear to be inconsistent. For example, higher residential densities are related to higher shares of non-motorized travel (e.g., cycling) [36, 37], while another study concluded that residential densities do not have a large influence on bicycle use [38]. Additionally, while green space was reported to be positively related to cycling [39, 40], others found no associations [25]. One major concern is that although it has been found that factors promoting or impeding cycling show significant spatial variation, most previous studies were based on the assumption that the relationship between individuals as well as environmental factors and cycling is spatially constant (i.e., built environment variables influence travel behavior in a similar manner everywhere) [41, 42]. However, the associations between cycling behavior and the natural and built environment characteristics might vary across areas [41, 42]. In addition, previous studies were mostly conducted within only one city or one region, whereas investigations covering a larger area (e.g., a whole country or several countries such as done in the study of [6]) are likely to result in more variation in environmental characteristics. More advanced statistical analyses, including interaction effects, may also uncover complex relationships between the environmental determinants and cycling behavior. For example, it is necessary to examine how the relationships between natural and built environmental determinants and cycling behavior may vary across areas, especially according to the urbanization level, as a previous review study has suggested [43]. This can offer a better insight into the role of natural and built environment characteristics for cycling across municipalities.

It is well-established that cycling behavior (e.g. duration and frequency) vary depending on people’s sociodemographic characteristics (e.g., age, gender, education level) [44–47] and across geographic scales [41, 48]. Although sociodemographic characteristics have been shown to be more strongly correlated with travel behavior than environmental factors [49, 50], urban form (e.g. municipality size) also seems to explain some variations in travel behavior. From a theoretical perspective, the socio-ecological model suggests a human-environment interplay [18]. It is therefore, necessary to examine urban form characteristics such as municipality size as a potential moderator of the relationship between natural and built environmental characteristics and cycling behavior. Such interactions may help to clarify inconsistent associations between environment and cycling.

To sum up, results concerning the associations between cycling duration and the natural and the built environment are contradictory [51], research on how natural and built environment characteristics relate to between-area variation in cycling duration is inconclusive, and whether the associations between these environment characteristics and cycling duration differ across settings (e.g., whether they are moderated by municipality size) [6] is unknown. The aim of the present research was to investigate the extent to which objectively measured natural and built environment characteristics contribute to differences in cycling (for all purposes) duration in adults (20–89 years) between 4-digit postal code areas (PC4 areas) and between municipalities across the Netherlands, and to explore interaction effects between environment characteristics and municipality size on cycling. We hypothesized that the associations between cycling duration and natural and the built environment characteristics would vary across areas and be potentially moderated by municipality size. To our knowledge, no previous study has investigated the hypothesized moderating effect of municipality size on the association between natural and the built environment and cycling.

## Methods

### Study design

**Fig. 1:**
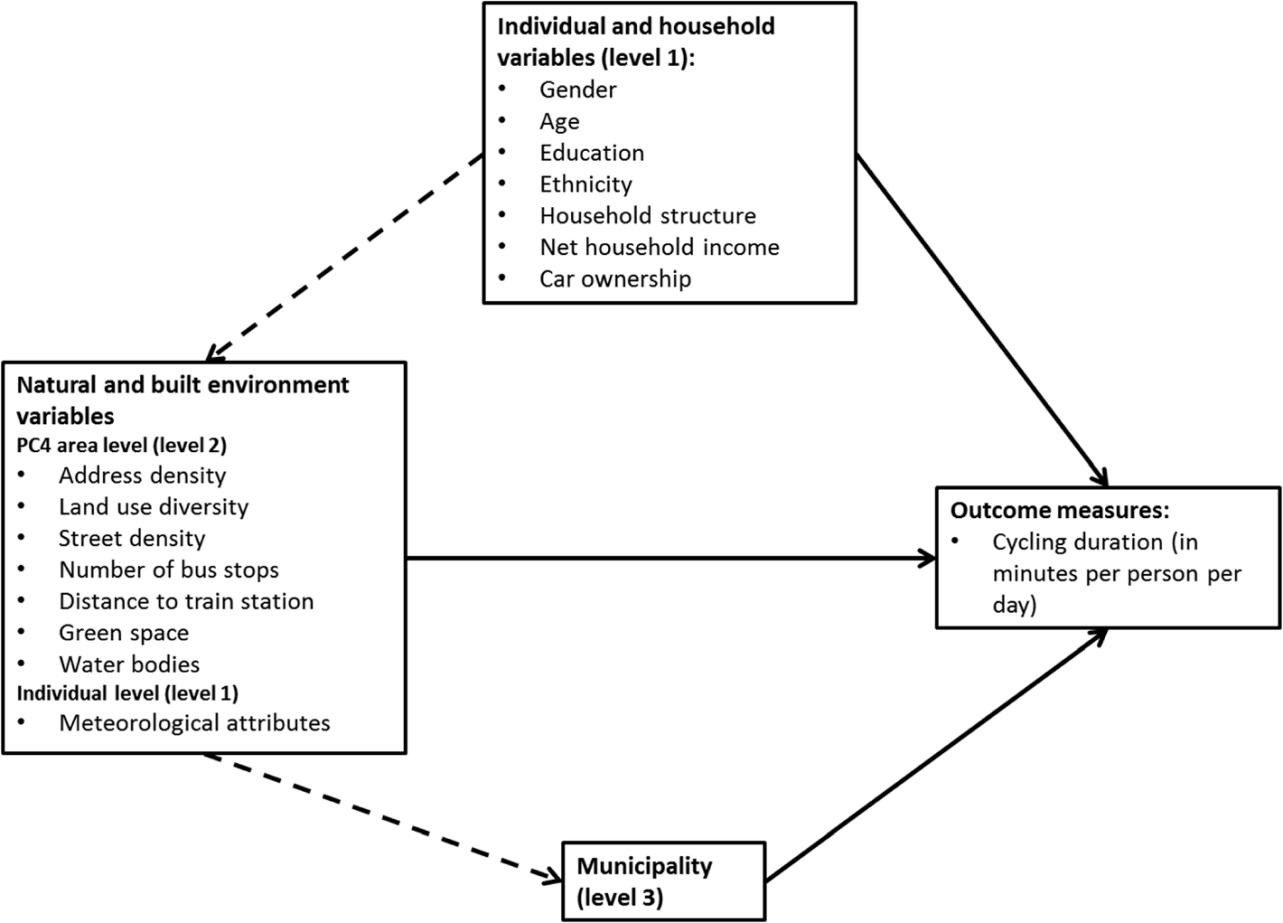
Conceptual model

This study was cross-sectional for the period 2010–2014 and dealt with adults aged 20–89 years residing in 3163 postal code areas (i.e., PC4 level) nested in 387 municipalities across the Netherlands. Figure 1 summarizes the underlying conceptual model.

### Travel survey data

Data were obtained from the Dutch National Travel Survey (NTS) for the period 2010–2014. The NTS is a continuous survey of approximately 40,000 individuals conducted annually by Statistics Netherlands [52]. Respondents report their transportation behavior by means of a travel diary for 1 day. For each trip, travel data include transportation modes, place of origin and destination, time of departure and arrival, and travel purpose. The sample is representative of the Dutch population. The respondents’ residential locations were geocoded on a PC4 level, which allowed data linkages with attributes describing the residential environment. Participants without postal code information (*n* = 708) were excluded from the research. This resulted in a final sample of 110,027 people aged 20–89 years, residing in 3163 PC4 areas with a mean number of respondents of 34 people (SD = 31), nested in 387 municipalities (level 3).

### Cycling duration

The outcome variable was total daily cycling duration in minutes per person. This included cycling for all purposes, i.e. travel-related as well as recreational cycling. Cycling duration was calculated based on the travel dairy data.

### Built environment variables

The selection of built environment measures was guided by the literature [29, 53]. The variables were calculated at the PC4 level using existing spatial data [54–56] for the year 2014. Address density refers to the total number of addresses divided by the PC4 area [56]. Land-use diversity is represented by a Shannon entropy index. A value of 0 refers to one land use class per area, and a value of 1 refers to an even distribution of all land use types per area [26, 57]. The operationalization considered the five most relevant land use types for residents’ daily activities, namely residential, commercial, industrial, and recreational areas, and public services (e.g., police station, hospital) [56]. Street density [58], distance to nearest train station [54], and number of bus stops per PC4 reflect transportation-related built environment measures. The latter also reflects competition between bicycle and public transportation.

**Fig. 2:**
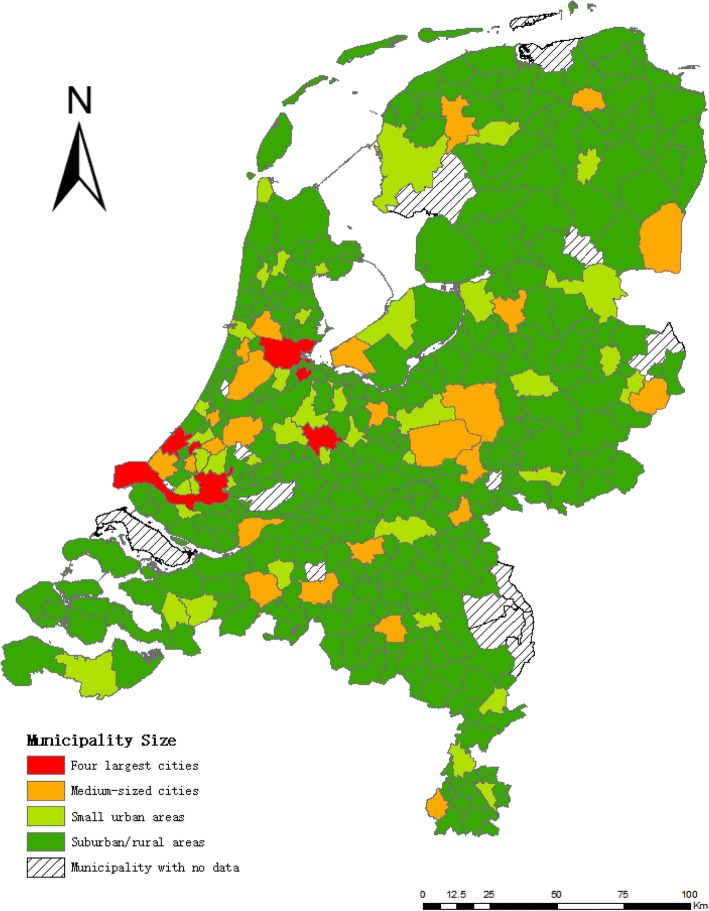
Distribution of four types of municipality size in the Netherlands. Municipality size was classified into four classes: the four largest cities, which have > 250,000 inhabitants (i.e., Amsterdam, Rotterdam, Den Haag, and Utrecht); medium-sized cities with 100,000–250,000 inhabitants; small urban areas with 50,000–100,000 inhabitants; and suburban/rural areas with < 50,000 inhabitants

Based on the population of each municipality, municipality size was classified into four classes: the four largest cities, which have > 250,000 inhabitants (i.e., Amsterdam, Rotterdam, Den Haag, and Utrecht); medium-sized cities with 100,000–250,000 inhabitants; small urban areas with 50,000–100,000 inhabitants; and suburban/rural areas with < 50,000 inhabitants (see Figure 2 in the Appendix 1) [59].

### Natural environment variables

Daily meteorological variables were collected from 33 weather stations across the Netherlands [60]. We obtained weather data from the weather station closest to each participant’s residential area for the day on which the travel diary was kept. We matched the trip date with daily measures of maximum air temperature (in °C), precipitation sum (in mm), and average wind speed (in m/s), all of which are frequently used measures [31, 42]. The proportion of green space (including agricultural and natural areas, man-made greenery (e.g., parks)) and water space per PC4 was abstracted from the most recent Dutch land use database for the year 2012 [61].

### Individual and household characteristics

Individual characteristics were obtained from the National Travel Survey. We categorized net household income per year into low (< €20,000), medium (€20,000–40,000), and high (>€40,000) [62]. Educational attainment was stratified into three categories: low (i.e., primary school and lower general secondary school), medium (i.e., upper-division secondary school), and high (i.e., college and university) [63]. We also controlled for numerous other demographic characteristics, including age, gender, ethnicity, household structure, and car ownership.

### Statistical analyses

Descriptive statistics were used to summarize the data, and Pearson correlation coefficients were used to test for multicollinearity among the covariates. Correlations < − 0.8 or > 0.8 are considered problematic [64].

To examine the associations between the built environment variables and cycling duration, we constructed multilevel regression models that allowed variables observed at different hierarchical levels (i.e., individuals nested in PC4 area, nested in municipalities). Unlike basic regressions, multilevel models can capture correlations that arise due to hierarchical data structures [65–67]. Furthermore, as the outcome variable (cycling duration) could not be negative, and showed an excess of zeros due to a relatively large share of respondents not reporting any cycling trips on the day of the survey, wrongly assuming that data were not censored to zero would have led to wrongly predicting a non-existent negative value. We therefore applied a multilevel Tobit regression model, which can better handle the dependent variables’ absence of negative values and excess of zero [68]. To facilitate the interpretation of the variance at both PC4 area level and municipality level, we calculated two intraclass correlation coefficients (ICC) [69]. The ICC refers to the proportion of total variance in the outcome that is attributable to the PC4 level and municipality level [70]. For example, the ICC in level 2 expresses the similarity in cycling duration between persons located in the same PC4. An ICC equal to 100% would imply that all people in a PC4 are have a similar cycling duration, while an ICC equal to 0% imply that people do not share any PC4 area related cycling duration.

Our multilevel regression models were restricted to random intercepts, because the average cluster size was small (i.e., on average 34 people were nested in each PC4 area and eight PC4 areas were nested in each municipality), resulting in reduced power for both random intercepts and slopes model [71].

We estimated the following models. First, we fitted a three-level random intercept model without explanatory variables (model 1). Second, model 1 was extended with individual and household variables (model 2). Third, model 3 also included both the natural and the built environment variables. Due to varying units, the continuous variables were standardized, and the most frequent category was used as the reference category. Subsequently, interactions between environmental factors and municipality size were tested in separate models, by adding the interaction term based on model 2, which resulted in a total of 10 interaction models. Significance was interpreted using the 95% confidence interval (CI). This interaction approach was based on previous interaction studies [72, 73]. All models were implemented in Stata 15.

## Results

### Descriptive statistics

**Fig. 3:**
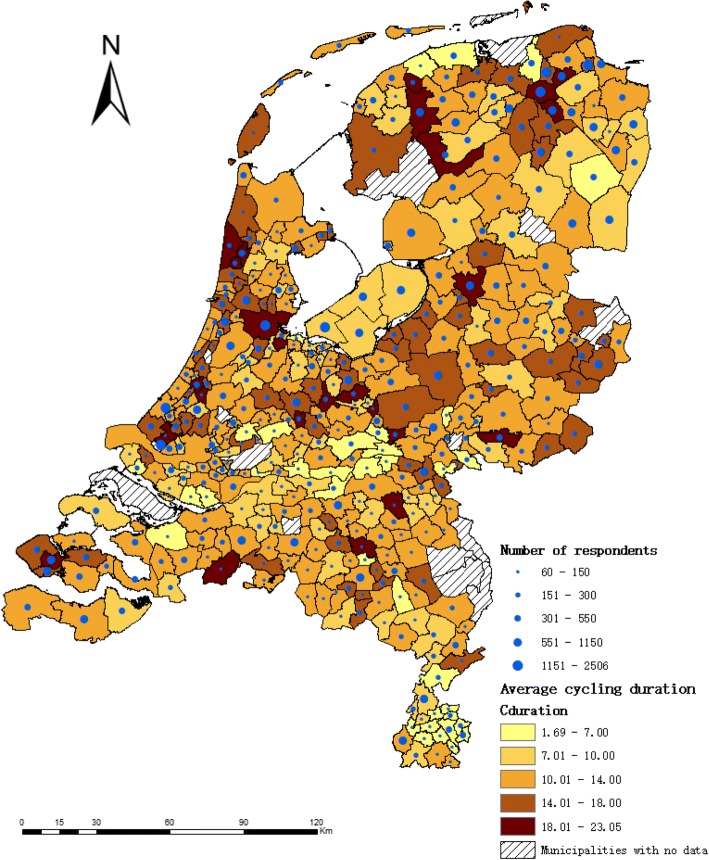
Distribution of average cycling duration (in minutes) and number of respondents in each municipality. Number of respondents in each PC4 area was represented as different sizes dots. Average cycling duration was presented in minutes

The sample comprised participants aged from 20 to 89 years, 51.7% were females. Most people aged 40–49 years (21.5%) and 50–59 years (20.8%). About 30.4% of the participants (*n* = 33,443) cycled for more than 1 min per day, the average daily cycling duration was 42.2 min per day. Among all participants, the average daily cycling duration was 12.8 min. Also, the variations between PC4 areas were larger (average duration = 11.86 min, SD = 10.86) than they were between municipalities (average duration = 11.81 min, SD = 3.77). The longest average cycling duration occurred in Groningen, a municipality in the north of the Netherlands (see Figure 3 in the Appendix 2). Further, descriptive statistics regarding cycling behavior, sociodemographic characteristics, natural and built environment characteristics, and different municipality sizes are presented in Table 1. Multicollinearity among the covariates was not a concern, as indicated by the Pearson correlations (see Table 5 in the Appendix 3).

**Table 1:** Descriptive statistics

Indicators	Measures	All participants (*N* = 110,027)	
		Mean(S.D.)	% per category
Dependent variables			
Cycling duration (in minutes)	≥0 min per day	12.8 (32.6)	
Individual and household variables			
Gender	Male		48.3%
	Female		51.7%
Age	20–29		12.8%
	30–39		16.0%
	40–49		21.5%
	50–59		20.8%
	60–69		17.1%
	70–79		9.1%
	80–89		2.7%
Household structure	Single-person household		17.1%
	Couple without children		36.8%
	Couple with children		40.4%
	Single parent with children		4.5%
Net household income	< €20,000		13.1%
	€20,000–40,000		42.8%
	>€40,000		44.2%
Education	Low		27.4%
	Medium		37.6%
	High		35.1%
Ethnicity	Dutch		94.4%
	Non-Dutch		5.6%
Car ownership	No car		10.8%
	1 car		52.6%
	2 or more cars		36.6%
Built environment variables			
Address density (1000 addresses per km^2^)		1.31 (1.54)	
Land use diversity		0.62 (0.21)	
Street density (km/km^2^)		16.30 (8.83)	
Number of bus stops		13 (10.58)	
Distance to train station (km)		6.79 (7.29)	
Natural environment variables			
Green space (%)		61.59 (23.34)	
Water bodies (%)		4.18 (6.52)	
Daily max. Air temperature (°C)		14.6 (7.2)	
Daily precipitation sum (mm)		4.1 (1.96)	
Daily average wind speed (m/s)		2.01 (4.33)	
Percentage of respondents in each municipality size	Four largest cities		8.6%
	Medium-sized cities		21.3%
	Small urban areas		17.3%
	Suburban/rural areas		52.7%

### Multilevel Tobit regression model to explain cycling duration

As shown in Table 2, associations between individual and household characteristics and cycling duration were all significant (Model 2) and remained significant after taking environmental characteristics into account (Model 3). The variance in cycling duration decreased from 3.5% (in model 2) to 3.4% (in model 3) at the PC4 area level, indicating that cycling duration variation between PC4 areas could be to a minor extent explained by natural and built environmental characteristics (see Table 2). Rather, an alternative reason for the minor difference could be the actual PC4 areas do not correspond with the boundaries that shape the relevant environment for cycling duration [70]. For municipalities, the variance in cycling duration remained at 2.2%, also after taking environmental characteristics into account.

**Table 2:** Results of the three-level Tobit regression model for cycling duration

	Model 1 (S.E.)	Model 2 (S.E.)	Model 3 (S.E.)
Intercept	−1.80*** (0.02)	−1.18*** (0.03)	−1.13*** (0.04)
Individual and household level			
Age (yrs.)			
20–29 (ref. = 40–49)		− 0.13*** (0.03)	− 0.14*** (0.03)
30–39		− 0.18*** (0.03)	− 0.19*** (0.03)
50–59		0.20*** (0.03)	0.20*** (0.03)
60–69		0.35*** (0.03)	0.36*** (0.03)
70–79		0.13*** (0.04)	0.13*** (0.04)
80–89		−0.77*** (0.06)	− 0.76*** (0.06)
Gender			
Man (ref. = Female)		−0.28*** (0.02)	− 0.28*** (0.02)
Education			
Lower (ref. = medium)		−0.08*** (0.02)	− 0.08*** (0.02)
Higher		0.19*** (0.02)	0.18*** (0.02)
Net household income			
< €20,000 (ref.= > €40,000)		−0.14*** (0.03)	− 0.14*** (0.03)
€20,000–40,000		−0.10*** (0.02)	−0.10*** (0.02)
Ethnicity			
Other (ref. = Dutch)		−0.83*** (0.04)	− 0.85*** (0.04)
Household structure			
Single-person household (ref. = Couple with children)		−0.56*** (0.03)	− 0.57*** (0.03)
Couple without children		−0.22*** (0.02)	−0.23*** (0.02)
Single parent with children		−0.43*** (0.04)	−0.43*** (0.04)
Car ownership			
No car (ref. = 1 car)		0.95*** (0.03)	0.93*** (0.03)
2 or more cars		−0.96*** (0.02)	−0.95*** (0.02)
Daily weather conditions			
Daily average wind speed (m/s)		−0.08*** (0.01)	−0.08*** (0.01)
Daily max. Air temperature (°C)		0.27*** (0.01)	0.27*** (0.01)
Daily precipitation sum (mm)		−0.07*** (0.01)	−0.08*** (0.01)
4-digit postal code zone level			
Address density (1000 addresses per km^2^)			0.09*** (0.21)
Land use diversity			−0.01 (0.01)
Street density (km/km^2^)			−0.02 (0.02)
Number of bus stops			0.02* (0.01)
Distance to train station (km)			−0.06** (0.02)
Percentage of green (%)			−0.04 (0.02)
Percentage of water (%)			−0.02 (0.01)
Level 1: individual and household			
Variance intercept ${\sigma}_1^2$	5.29 (0.05)	4.98 (0.04)	4.98 (0.04)
Level 2: 4-digit postal code			
Variance intercept ${\sigma}_2^2$	0.11 (0.01)	0.07 (0.01)	0.06 (0.01)
Level 2: ICC	4.4%	3.5%	3.4%
Level 3: Municipality			
Variance intercept ${\sigma}_3^2$	0.14 (0.02)	0.11 (0.01)	0.11 (0.01)
Level 3: ICC	2.5%	2.2%	2.2%

Sig. Codes: *p ≤ 0.050; **p ≤ 0.010; ***p ≤ 0.001

The results of model 3 suggest that the built environment variables were largely associated with cycling duration, also when individual and household variables were controlled for. Respondents living in PC4 areas with a higher address density, more bus stops, and shorter distance to the nearest train station tended to cycle longer. A higher temperature was also positively related to cycling duration. Wind speed and precipitation as well as percentage of green showed an inverse correlation with cycling duration. No significant associations were found between cycling duration and the other natural and built environment variables, such as land-use diversity, street density, and water bodies.

Ten interactions between natural and built environment and municipality size were significant (Tables 3 and 4). Several associations between environmental characteristics and cycling were weaker in small urban or rural areas than in urbanized areas. Specifically, the positive associations between address density and street density and cycling duration in larger cities were smaller or even negative in small urban areas. This may explain the nonsignificant association between street density and cycling duration when the interaction effects are not considered in model 3. In contrast, the positive association between number of bus stops and cycling duration was weaker in the four largest cities and the medium-sized cities compared to small urban and rural areas. Further, the negative association between distance to train station and cycling duration was strongest in large cities, compared to less urbanized municipalities. More green was inversely related to cycling duration, and this association was most pronounced in the four largest cities.

**Table 3:** Associations of built environment attributes, municipality size, and built environment × municipality size interactions with cycling duration

Municipality size	Address density (1000 addresses per km^2^) (AD)		Land use diversity (LD)		Street density (km/km^2^) (SD)		Number of bus stops (BS)		Distance to train station (km) (DT)	
		β (95%CI)		β (95%CI)		β (95%CI)		β (95%CI)		β (95%CI)
Four largest cities (M1) (ref. = M4)	AD	0.208***(0.11, 0.31)	LD	−0.018 (− 0.05,0.01)	SD	0.108***(0.06, 0.15)	BS	0.066***(0.03, 0.10)	DT	−0.08***(− 0.12,-0.04)
	M1	−0.299 (− 0.64, 0.05)	M1	0.035 (− 0.30,0.37)	M1	− 0.105 (− 0.46, 0.25)	M1	0.040 (− 0.30, 0.38)	M1	−0.196 (− 0.56, 0.17)
	AD×M1	−0.081 (− 0.19, 0.03)	LD × M1	0.024 (− 0.05,0.10)	SD × M1	−0.037 (− 0.15, 0.07)	BS × M1	−0.136***(− 0.21,-0.06)	DT × M1	−0.398* (− 0.73,-0.06)
Medium-sized cities (M2)	AD	0.208***(0.11, 0.31)	LD	−0.018 (− 0.05,0.01)	SD	0.108***(0.06, 0.15)	BS	0.066***(0.03, 0.10)	DT	−0.08***(− 0.12,-0.04)
	M2	−0.007 (− 0.15, 0.16)	M2	0.137 (− 0.01,0.28)	M2	0.060 (− 0.09, 0.21)	M2	0.135 (− 0.01, 0.28)	M2	0.033 (− 0.13, 0.20)
	AD×M2	0.101 (−0.22, 0.02)	LD × M2	−0.027 (− 0.08,0.03)	SD × M2	− 0.069 (− 0.15, 0.01)	BS × M2	−0.065* (− 0.12,-0.01)	DT × M2	−0.152 (− 0.36, 0.06)
Small urban areas (M3)	AD	0.208***(0.11 ,0.31)	LD	−0.018 (− 0.05,0.01)	SD	0.108***(0.06, 0.15)	BS	0.066***(0.03, 0.10)	DT	−0.08***(− 0.12,-0.04)
	M3	−0.054 (− 0.18, 0.08)	M3	0.046 (− 0.08,0.17)	M3	− 0.014 (− 0.14, 0.11)	M3	0.042 (− 0.08, 0.17)	M3	0.012 (− 0.11, 0.14)
	AD×M3	− 0.209** (− 0.36,-0.06)	LD × M3	0.010 (− 0.05,0.07)	SD × M3	−0.108** (− 0.19,-0.03)	BS × M3	−0.064 (− 0.14, 0.01)	DT × M3	−0.034 (− 0.16, 0.09)

All models adjusted for age, gender, income, education, ethnicity, household structure, and number of cars in the household

M4 = Suburban/rural areas are the reference category

Sig. Codes: *p ≤ 0.050; **p ≤ 0.010; ***p ≤ 0.001

**Table 4:** Associations of natural environment attributes, municipality size, and natural environment × municipality size interactions with cycling duration

Municipality size	Daily average wind speed (m/s) (W)		Daily max air temperature (°C) (T)		Daily precipitation sum (mm) (R)		Percentage of green (%) (G)		Percentage of water (%) (WA)	
		β (95%CI)		β (95%CI)		β (95%CI)		β (95%CI)		β (95%CI)
Four largest cities (M1) (ref. = M4)	W	−0.172***(− 0.197,-0.146)	T	0.320***(0.30, 0.34)	R	−0.105***(− 0.13,-0.08)	G	− 0.082***(− 0.12,-0.04)	WA	−0.025 (− 0.06, 0.01)
	M1	0.067 (−0.27, 0.41)	M1	0.049 (−0.29, 0.39)	M1	0.037 (−0.30, 0.37)	M1	−0.308 (− 0.66, 0.04)	M1	0.035 (− 0.30, 0.37)
	W × M1	0.041 (−0.02, 0.10)	T × M1	−0.175***(− 0.24,-0.11)	R × M1	−0.012 (− 0.05, 0.07)	G × M1	−0.187***(− 0.30,-0.08)	WA × M1	0.028 (− 0.03, 0.09)
Medium-sized cities (M2)	W	−0.172***(− 0.197,-0.146)	T	0.320***(0.30, 0.34)	R	−0.105***(− 0.13,-0.08)	G	−0.082***(− 0.12,-0.04)	WA	−0.025 (− 0.06, 0.01)
	M2	0.146 (−0.001, 0.29)	M2	0.148*(0.00, 0.29)	M2	0.142 (−0.003, 0.29)	M2	0.056 (−0.09, 0.21)	M2	0.147* (0.002, 0.29)
	W × M2	−0.030 (− 0.02, 0.08)	T × M2	− 0.058** (− 0.10,-0.15)	R × M2	−0.030 (− 0.02, 0.08)	G × M2	0.001 (− 0.07, 0.07)	WA × M2	−0.066 (− 0.13, 0.002)
Small urban areas (M3)	W	−0.172***(− 0.197,-0.146)	T	0.320***(0.30, 0.34)	R	−0.105***(− 0.13,-0.08)	G	−0.082***(− 0.12,-0.04)	WA	−0.025 (− 0.06, 0.01)
	M3	0.045 (− 0.08, 0.17)	M3	0.055 (− 0.07, 0.18)	M3	0.050 (− 0.07, 0.17)	M3	− 0.004 (− 0.13, 0.12)	M3	0.050 (− 0.07, 0.17)
	W × M3	0.015 (− 0.04, 0.07)	T × M3	−0.047* (− 0.09, 0.0001)	R × M3	0.058* (0.01, 0.11)	G × M3	0.044 (− 0.04, 0.12)	WA × M3	0.028 (− 0.04, 0.10)

All models adjusted for age, gender, income, education, ethnicity, household structure, and number of cars in the household

M4 = Suburban/rural areas are the reference category

Sig. Codes: *p ≤ 0.050; **p ≤ 0.010; ***p ≤ 0.001

## Discussion

### Key findings

People living in areas with a high address density, more bus stops, and shorter distance to train station cycled longer. Further, cycling duration was positively related to higher temperatures, whereas rain and wind speed were negatively associated with cycling duration. Water bodies did not have a significant relation to cycling duration. Significant interactions of municipality size with built environment characteristics were found. Increased street density and address density appeared to be less cycling-promotive in small urban areas compared to medium or large cities. On the other hand, the positive association between number of bus stops and cycling duration was weaker in the four largest cities and in medium-sized cities compared to small urban and rural areas. This suggests that relations between environmental characteristics and cycling may be dependent on other circumstances (which differ between highly urbanized and less urbanized areas) and are thus context-specific.

### Explanation of key findings

When natural and built environment characteristics were included in the models, the variance of cycling duration between PC4 areas declined slightly, but variations at the municipality level could not be explained by environmental characteristics at all. One plausible reason is that because these built environment variables are measured at the PC4 area level, they are inherently more capable of explaining the variance change at the PC4 level than at the municipality level. Another possible explanation is that the low variability in urban design may be typical of Dutch urban areas. The Netherlands is a high-density country with a very good cycling infrastructure and a flat topography. A Dutch walking study also came up with similar findings [72].

Consistent with previous studies [39, 74], an association was found between built environment variables and cycling duration: people living in areas with a high address density, more bus stops, and a shorter distance to a train station cycled longer. This may be because, for example, popular destinations, like the city center also have many bus stops. Likewise, people would also be more likely to undertake cycling activities in the city center. In addition, previous studies found that when the distance between a residence and a train station is 1.5–3.7 km, bicycle use increases [24, 75]. In the Netherlands, active transportation modes like cycling play a key role in access to and egress from public transportation (e.g., bus stops, train stations): Nearly 50% of all trips between home and train station were conducted by bike [14].

Contrary to previous findings [76], in our study land use diversity was not significantly associated with cycling duration. The contrary evidence suggests that mixed-use development in high-density cities may not always have the expected, positive effect on cycling duration. This may be because a high level of land use diversity is associated with heavy traffic, which might eventually weaken people’s motivation to cycle [39]. Also, the non-significant association between street density and cycling duration in this study could be explained by a relatively high level of connectivity, which is in line with previous studies [77]. The percentage of green and water surface was negatively related to cycling duration, which is also in line with other studies [47, 78]. This negative effect suggests that whereas people living in attractive residential areas (with green and water areas) tend to remain in and around their houses or gardens, people living in less attractive areas, with relatively little green and/or water, report more cycle trips. Another possible explanation is that most direct routes are through areas with less green and water, as most individuals want to get to their destination as quickly and easily as possible [47].

Weather variables were significantly related to cycling duration, which confirms previous Dutch studies [34, 33, 42]. High daily precipitation total and high average wind speed had negative effects on cycling, whereas cyclists were more keen to ride when there were high levels of sunshine and high temperatures, as pleasant weather stimulated cycling for recreation [33].

Most of the built environment characteristics tested had significant main effects on cycling duration, independent of municipality size [79]. A key finding of the present study was that several associations between built environment characteristics and cycling were stronger in urbanized areas than in small urban or rural areas. Specifically, interactions indicated that the association of address density and street density with cycling duration was weaker in small urban areas compared to larger cities. On the other hand, the positive association between number of bus stops and cycling duration was weaker in the four largest cities and the medium-sized cities compared to small urban and rural areas. One interpretation is that more bus stops means more motorized vehicles on the road, which increases the risk of collisions between bicycles and motorized vehicles, especially in large urban areas [7]. Another possible explanation is that there is competition between public transportation and bicycle use, which is confirmed by previous research [80]. In addition, the negative association between distance to the nearest train station and cycling duration was stronger in the four largest cities, indicating that a shorter distance to the nearest train station encourages people to cycle more. Regarding the daily maximum temperature, the association with cycling duration was weaker in the four largest cities than in rural areas. A possible explanation is that most Dutch cities have a substantial urban heat island (UHI) that is significantly warmer than the surrounding rural areas due to human activities [81].

### Strengths and limitations

The study results need to be interpreted in light of some limitations. First, whereas the NTS data for the period 2010–2014 were used, most built environment variables describe the situation in 2014, and for green space and water bodies we used data from 2012. However, the built environment characteristics used are not expected to have changed much over a couple of years. Second, bicycle use was not separately analyzed in terms of trip purpose. However, associations between natural and built environment characteristics and cycling duration differ by trip purpose [82]. For example, increasing the tree coverage could increase recreational cycling. It would be interesting for future studies to examine cycling duration for different trip purposes separately. Notably, the relationships between natural and built environment characteristics and cycling duration are complex, and the further investigation of those relationships is needed. Also, future studies should consider not only individual or built environment characteristics associated with cycling behavior, but also personal motivation, travel mode preferences, or mental health, which may vary regarding environmental awareness and/or attitudes toward cycling.

This paper has several key strengths. First, as far as we are aware, this is the first study to examine the extent to which natural and built environment characteristics contribute to inter-PC4 area and inter-municipality differences in cycling behavior in a national Dutch context. Also to the best of our knowledge, no previous studies have investigated municipality size as a moderator between built environment and cycling duration. The uniform and good quality data collocation across years for the whole country reduced the bias during the research and increased the potential for generalizing the results. Second, due to recall biases, measuring aspects of natural and built environment subjectively (e.g., self-report methods) may not accurately assess the association between cycling behavior and the actual natural and built environment characteristics [83, 84]. Therefore, in this study, objective measurements of the natural and built environment were made, which could provide an understanding of how the built environment is constructed regarding policy and urban planning. Third, the hierarchical structure of data, ranging from the individual to the municipality level, was taken into account to correct for possible biases and enable an exploration of variables at different data levels [65].

Because we focused on the environmental variability between PC4 areas and municipalities, the within-PC4 variability was not considered. If natural and built environment characteristics were measured at a more fine-grained area level, this could increase the variation and the understanding of individual travel behavior. However, it is also important to understand the between-area variation (both PC4 area and municipality level), as policies are mainly based on between-municipality variances. Nonetheless, we maximized variation in natural and built environment characteristics, by including many different PC areas across the whole of the Netherlands, including urban and rural areas. Finally, the interaction effects may suggest that relations between environmental characteristics and cycling duration are context-specific (i.e., dependent on circumstances that differ between urbanized and suburban/rural areas). Other countries may benefit from examining Dutch transportation policies, in order to determine whether there are opportunities to adopt or adapt some of these within their own transportation policy environment.

## Conclusions

Higher address density, more bus stops, and shorter distance from home to the nearest train station were positively related to cycling duration. In addition, significant interaction effects suggest that municipality size may moderate the association between environmental characteristics and cycling duration. Our findings need to be replicated in other countries to gain more insight into the interplay between environment and municipality size.

## Appendix 3

### Test for correlation between built environment variables

**Table 5:** Pearson correlation between built environment variables (*N* = 110,027)

	Address density	Land use diversity	Street density	Number of bus stops	Distance to train station
Address density	1	−.150**	.709**	.035**	−.311**
Land use diversity	–	1	−.226**	.103**	.092**
Street density	–	–	1	−.007*	−.371**
Number of bus stops	–	–	–	1	−.071**
Distance to train station	–	–	–	–	1

Sig. codes: **p* ≤ 0.050; ***p* ≤ 0.010

## Abbreviations

AD: Address density
BS: Number of bus stops
CI: Confidence interval
DT: Distance to train station
ICC: Intraclass correlation coefficients
LD: Land-use diversity
NTS: National Travel Survey
PC4 areas: 4-digit postal code areas
SD: Street density

## Footnotes

1 Heerlen: 45.53 km^2^, 1929 people per km^2^.

2 Groningen: 83.75 km^2^, 2572 people per km^2^.
